# Crystal structure of the N-myristoylated lipopeptide-bound MHC class I complex

**DOI:** 10.1038/ncomms10356

**Published:** 2016-01-13

**Authors:** Daisuke Morita, Yukie Yamamoto, Tatsuaki Mizutani, Takeshi Ishikawa, Juri Suzuki, Tatsuhiko Igarashi, Naoki Mori, Takashi Shiina, Hidetoshi Inoko, Hiroaki Fujita, Kazuhiro Iwai, Yoshimasa Tanaka, Bunzo Mikami, Masahiko Sugita

**Affiliations:** 1 Laboratory of Cell Regulation, Institute for Virus Research, Kyoto University, 53 Kawahara-cho, Shogoin, Sakyo-ku, Kyoto 606-8507, Japan; 2 Laboratory of Cell Regulation and Molecular Network, Graduate School of Biostudies, Kyoto University, Yoshida-Konoe-cho, Sakyo-ku, Kyoto 606-8501, Japan; 3 Department of Molecular Microbiology and Immunology, Graduate School of Biomedical Sciences, Nagasaki University, 1-12-4 Sakamoto, Nagasaki 852-8523, Japan; 4 Center for Human Evolution Modeling Research, Primate Research Institute, Kyoto University, Inuyama, Aichi 484-8506, Japan; 5 Center for Emerging Virus Research, Institute for Virus Research, Kyoto University, 53 Kawahara-cho, Shogoin, Sakyo-ku, Kyoto 606-8507, Japan; 6 Laboratory of Chemical Ecology, Division of Applied Life Sciences, Graduate School of Agriculture, Kyoto University, Kitashirakawa-Oiwake-cho, Sakyo-ku, Kyoto 606-8502, Japan; 7 Division of Basic Medical Science and Molecular Medicine, Department of Molecular Life Science, Tokai University School of Medicine, Isehara, Kanagawa 259-1143, Japan; 8 Department of Molecular and Cellular Physiology, Graduate School of Medicine, Kyoto University, Yoshida-konoe-cho, Sakyo-ku, Kyoto 606-8501, Japan; 9 Center for Bioinformatics and Molecular Medicine, Graduate School of Biomedical Sciences, Nagasaki University, 1-14 Bunkyo-machi, Nagasaki 852-8521, Japan; 10 Laboratory of Applied Structural Biology, Division of Applied Life Sciences, Graduate School of Agriculture, Kyoto University, Gokasho, Uji, Kyoto 611-0011, Japan

## Abstract

The covalent conjugation of a 14-carbon saturated fatty acid (myristic acid) to the amino-terminal glycine residue is critical for some viral proteins to function. This protein lipidation modification, termed N-myristoylation, is targeted by host cytotoxic T lymphocytes (CTLs) that specifically recognize N-myristoylated short peptides; however, the molecular mechanisms underlying lipopeptide antigen (Ag) presentation remain elusive. Here we show that a primate major histocompatibility complex (MHC) class I-encoded protein is capable of binding N-myristoylated 5-mer peptides and presenting them to specific CTLs. A high-resolution X-ray crystallographic analysis of the MHC class I:lipopeptide complex reveals an Ag-binding groove that is elaborately constructed to bind N-myristoylated short peptides rather than prototypic 9-mer peptides. The identification of lipopeptide-specific, MHC class I-restricted CTLs indicates that the widely accepted concept of MHC class I-mediated presentation of long peptides to CTLs may need some modifications to incorporate a novel MHC class I function of lipopeptide Ag presentation.

## Abstract (web summary)

Lipid antigens have been added to the antigenic repertoire in recent years. Here, the authors have identified Mamu-B*098 as a lipopeptide antigen presenting molecule and using structural and biochemical analysis have shown that it has a different antigen binding pocket to previously analysed proteins.

## Graphical abstract

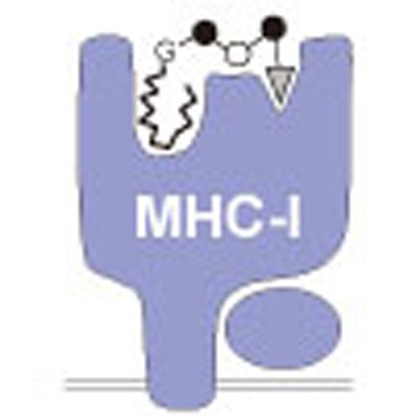

Research carried out over the past three decades has unravelled the molecular and structural bases of cell-mediated immunity, resulting in the establishment of a central paradigm for antigen (Ag) presentation in which major histocompatibility complex (MHC) class I and class II molecules bind peptide Ags and present them to CD8^+^ and CD4^+^ T cells, respectively, bearing clonotypic αβ T-cell receptors (TCRs)^1^. The repertoire of Ags targeted by αβ T cells has been further expanded to include lipid Ags, such as fatty acids and glycolipids, which are captured and presented by non-MHC-encoded group 1 CD1 molecules^2^. These two arms of the adaptive immune system, one directed against peptides and the other against lipids, may function cooperatively for effective host defense against microbial infections. Indeed, both peptide-specific and lipid-specific cytotoxic T lymphocytes (CTLs) have been identified as having the potential to eliminate *Mycobacterium tuberculosis*-infected cells^3^.

Unlike bacteria, viruses do not produce their own lipids; however, some viral proteins are lipid modified by using the host cellular machinery^4^,^5^,^6^. For example, human and simian immunodeficiency viruses (HIV and SIV) borrow host-derived N-myristoyltransferase and its substrate myristoyl-CoA, to couple a 14-carbon saturated fatty acid (myristic acid) to the N-terminal glycine residue of the Nef protein. This lipidation process, referred to as N-myristoylation, is essential for the Nef protein to downregulate the cell surface expression of MHC class I molecules, thereby inhibiting the efficient induction of virus-specific CTLs^7^,^8^. On the other hand, the findings of our recent studies have indicated that the adaptive immune system is equipped with CTLs capable of sensing the lipid modification reaction of the Nef protein^9^,^10^. The rhesus macaque CD8^+^ CTL line 2N5.1 specifically recognizes the N-myristoylated 5-mer peptide (C14-Gly-Gly-Ala-Ile-Ser; C14nef5) derived from the SIV Nef protein. Furthermore, N-myristoylated Nef peptide-specific T cells were found to expand significantly in the peripheral blood of SIV-infected monkeys and the plasma viral load in the infected monkeys correlated reciprocally with the number of circulating lipopeptide-specific T cells. These findings suggest that besides peptides (presented by MHC class I) and lipids (presented by group 1 CD1), lipopeptides may comprise a novel chemical class of Ags targeted by CTLs with implications in the host defense against viral infection; however, the identity of the lipopeptide Ag-presenting molecules currently remains unknown.

In the present study, we show that the classical MHC class I-encoded protein Mamu-B*098 captures C14nef5 and interacts with specific TCRs, thereby supporting the recognition of C14nef5 by 2N5.1 T cells. The capacity of the Mamu-B*098 protein to function as a lipopeptide Ag-presenting molecule is further substantiated by a high-resolution X-ray crystallographic analysis of the trimeric complex of B*098 heavy chains, β2-microglobulin (β2m) and C14nef5. The myristoyl group of the lipopeptide is accommodated in the relatively large B pocket lined with hydrophobic amino acid residues and the carboxy-terminal serine residue is anchored at the small F pocket via multiple hydrogen bonds, leaving the second and fourth amino acid residues exposed externally. Furthermore, a conduit between A and B pockets in Mamu-B*098 is spatially disconnected, making the A pocket isolated and unoccupied. These structural features have not been recognized previously for other MHC class I molecules that bind prototypic 9-mer peptides. Taken together, these results suggest that MHC class I molecules may have evolved to include those binding N-myristoylated short peptides, thereby assisting CTLs to specifically sense the N-myristoylation of viral proteins.

## Results

### Identification of a lipopeptide Ag-presenting molecule

**Figure 1:**
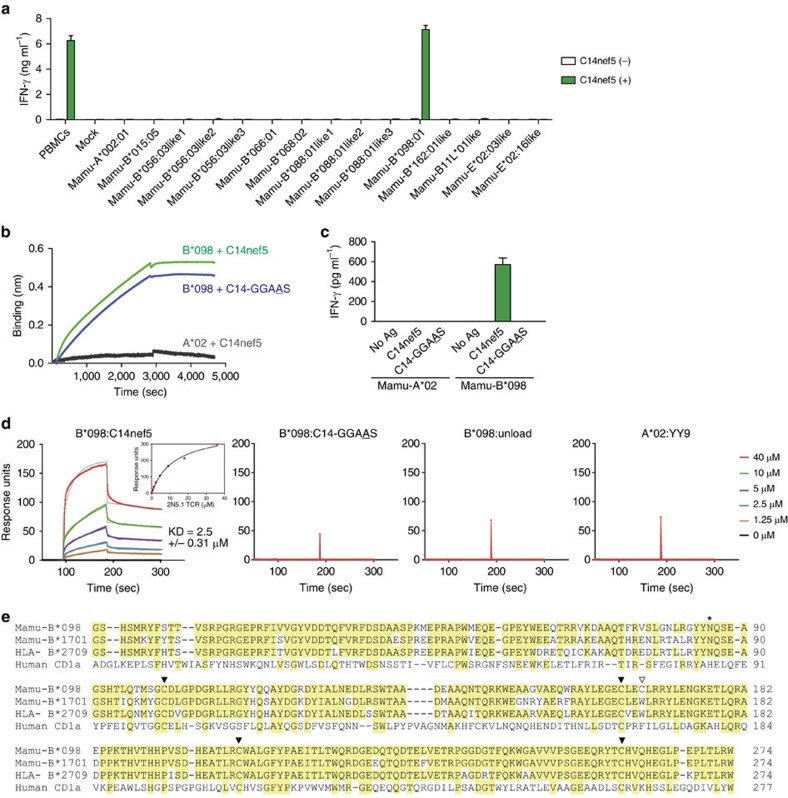
Identification of Mamu-B*098 as a lipopeptide Ag-presenting molecule. (**a**) LLC-MK2 cells transfected with each of the rhesus MHC class I genes were tested for their ability to present C14nef5 to 2N5.1. Only LLC-MK2 cells expressing Mamu-B*098 stimulated 2N5.1 to produce IFN-γ in the presence of C14nef5. PBMCs obtained from a donor with the potential to present C14nef5 to 2N5.1 were used as a positive control. Experiments were performed in triplicate. Mean values with s.e.m. are shown. (**b**) The binding of lipopeptide Ags to recombinant β2m-linked Mamu-B*098 and Mamu-A*02 proteins was monitored by biolayer interferometry. Representative data from three independent experiments are shown. Mamu-A*02, a rhesus MHC class I allele known to bind 9-mer peptides, was used as a negative control. (**c**) Plate-coated MHC class I molecules were tested for their ability to present lipopeptide Ags to 2N5.1. The Ag-specific T-cell response was assessed by measuring the amount of IFN-γ released into the culture medium. Mean values with s.e.m. are shown. (**d**) The high-affinity interaction of the 2N5.1 TCR with C14nef5-loaded Mamu-B*098 was demonstrated by SPR-binding assays, whereas no interaction was detected with C14-GGAAS-loaded and unloaded Mamu-B*098 molecules, as well as YY9 peptide-loaded Mamu-A*02 molecules. The original data (coloured as indicated) are depicted with the curve fit (grey) overlaid. The steady-state affinity plot (inset) is also shown. Three independent experiments were performed and mean equilibrium-dissociation constant (*K*_D_) values ±s.d. are shown. (**e**) Amino acid sequences of Mamu-B*098, HLA-B*27:09, Mamu-B*17:01 and human CD1a are shown. The α1 and α2 domains of Mamu-B*098 exhibit 81.1%, 80.6% and 20.6% sequence homology to the corresponding domains of HLA-B27, Mamu-B*17 and human CD1a, respectively. Solid and open triangles indicate paired and unpaired cysteine residues, respectively. The residue for N-glycosylation is indicated with an asterisk.

The recognition of C14nef5 by 2N5.1 requires the presence of irradiated peripheral blood mononuclear cells (PBMCs)^9^, which suggests that PBMCs express a molecule capable of binding C14nef5 and presenting it to 2N5.1. To obtain an insight into its molecular identity, we generated several hundred monoclonal antibodies that recognized rhesus PBMCs and tested their abilities to block the presentation of C14nef5 to 2N5.1. Two blocking monoclonal antibodies, MB217 and MB226, were isolated (Supplementary Fig. 1a) and a series of biochemical and cellular analyses revealed that MB217 specifically recognized rhesus β2m, whereas MB226 reacted broadly with rhesus MHC class I molecules (Supplementary Fig. 1b–e). As β2m-associated MHC class I molecules may restrict the recognition of C14nef5 by 2N5.1, we isolated an array of rhesus MHC (Mamu) class I complementary DNA clones from a donor with the potential to present C14nef5 to 2N5.1 and transfected each cDNA clone into the monkey kidney epithelial cell line LLC-MK2. The ability of the cell transfectants to present C14nef5 to 2N5.1 was then examined. None of the cell transfectants exhibited the Ag presentation capacity, except those expressing Mamu-B*098, which stimulated 2N5.1 cells to produce interferon-γ (IFN-γ) in the presence of C14nef5, but not in its absence (Fig. 1a). These results strongly suggested that the Mamu-B*098 protein may function as a lipopeptide Ag-presenting molecule.

To address this possibility directly, cell-free assays were performed with a soluble recombinant Mamu-B*098 protein. Using a biolayer interferometry technique, the physical interactions between C14nef5 and a layer of immobilized protein on the biosensor tip were demonstrated for Mamu-B*098 but not for the control protein Mamu-A*02 (Fig. 1b). Furthermore, plate-coated Mamu-B*098, but not Mamu-A*02, was sufficient for stimulating 2N5.1 to produce IFN-γ in response to C14nef5 (Fig. 1c). The non-antigenic mutant, C14-GGAAS, in which the fourth isoleucine residue was replaced by alanine, bound to B*098 (Fig. 1b) but failed to stimulate 2N5.1 even in the presence of Mamu-B*098 (Fig. 1c), suggesting that the isoleucine residue served as a T-cell epitopic determinant. Furthermore, surface plasmon resonance (SPR)-binding experiments revealed that a soluble TCR αβ dimer derived from 2N5.1 interacted with C14nef5-loaded, but not with C14-GGAAS-loaded, Mamu-B*098, with an affinity that was similar to that for peptide-specific TCR–MHC interactions (Fig. 1d)^11^. Taken together, these results provide compelling evidence that the Mamu-B*098 protein functions as a lipopeptide Ag-presenting molecule capable of binding C14nef5 and presenting it to T cells bearing specific αβ TCRs.

### Structure of Mamu-B*098

The amino acid sequence of Mamu-B*098 is very similar to those of conventional MHC class I molecules, such as Mamu-B*17 in rhesus macaques and HLA-B27 in humans, which mediate the presentation of 8- to 10-mer peptides to T cells (Fig. 1e), and provides no reasonable insight into how the MHC class I protein binds N-myristoylated 5-mer peptides that are structurally and chemically distinct from prototypic MHC class I-presented peptides.

To elucidate the molecular basis underlying lipopeptide Ag presentation, we sought to clarify the high-resolution X-ray crystal structure of the Mamu-B*098:C14nef5 complex. The ectodomain of the Mamu-B*098 heavy chain and rhesus β2m were produced in *Escherichia coli* as inclusion bodies and the purified recombinant proteins were refolded in the presence of C14nef5. In our initial attempts, proper refolding occurred inefficiently, which was most probably due to aberrant disulfide bond formation involving a cysteine residue at position 167 (Fig. 1e, shown with an open arrowhead). The introduction of the cysteine to serine mutation at this position resulted in improved refolding efficiency. This mutation affected neither the overall secondary structure nor the ability of Mamu-B*098 to present C14nef5 to 2N5.1 (data not shown). Thus, the amino acid substitution mutant of Mamu-B*098 was used for crystallization.

**Figure 2:**
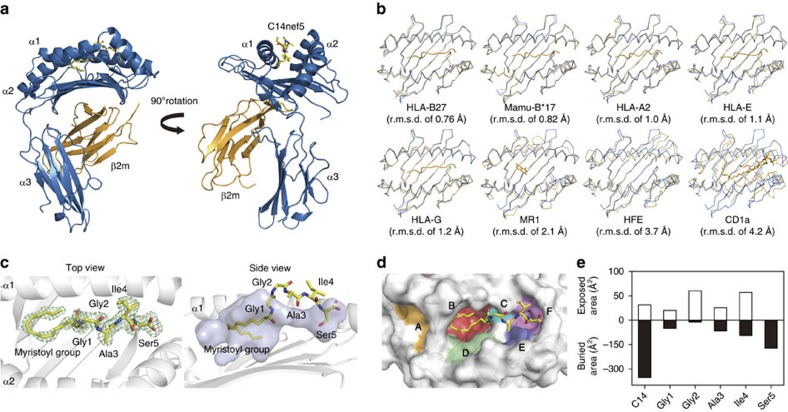
The overall structure of the Mamu-B*098 complex. (**a**) Two views of the trimer complex of the ectodomain of Mamu-B*098 heavy chains (blue), β2m (orange) and C14nef5 (yellow) are shown. (**b**) Superimposed images of the α1 and α2 domains of Mamu-B*098 (blue) with those of MHC class I and MHC class I-like molecules (orange) are shown. (**c**) C14nef5 binding to Mamu-B*098 is demonstrated by a 2Fo-Fc map (green mesh) contoured at 0.8*σ* (left). The bound lipopeptide accommodated in the semi-transparent Ag-binding cavity is shown (right). (**d**) The surface of the Ag-binding groove with pockets A through F is shown. (**e**) Solvent accessible surfaces of C14nef5 captured by Mamu-B*098 are shown for the myristoyl group and each of the amino acid residues. Filled and unfilled bars indicate buried and exposed areas, respectively.

We determined the crystal structure of the Mamu-B*098:C14nef5 complex at a resolution of 1.76 Å (Table 1 and Supplementary Fig. 2). The overall structure of Mamu-B*098 was almost indistinguishable from that of other MHC class I molecules, which were characterized by two semisymmetrical domains, α1 and α2, which formed a β-sheet platform topped by two semiparallel α-helices (Fig. 2a)^12^. The α1/α2-fold of Mamu-B*098 exhibited a high degree of structural similarity with those of HLA-B27 and Mamu-B*17 with root-mean-square deviations (r.m.s.d.) of 0.76 and 0.82 Å, respectively^13^,^14^, and the groove width, as measured by the distance between the opposing α1 and α2 helices, was similar among the MHC-B molecules (Fig. 2b). Continuous electron density corresponding to C14nef5 was observed in the Ag-binding groove, thus providing direct evidence for the capacity of Mamu-B*098 to bind the lipopeptide Ag (Fig. 2c).

As for other MHC class I molecules, six pocket structures, designated A through F, were identified in the Ag-binding groove of Mamu-B*098 (Fig. 2d). The myristoyl group and C-terminal serine residue (Ser5) were buried deeply in the groove and interacted primarily with the B pocket and F pocket, respectively, forcing the central three residues of the peptide chain, in particular Gly2 and Ile4, to protrude out of the groove and into the solvent (Fig. 2c,e). These structural features were consistent with the prediction deduced from the T-cell assays^10^.

### Accommodation of the myristoyl group in the B pocket

**Figure 3:**
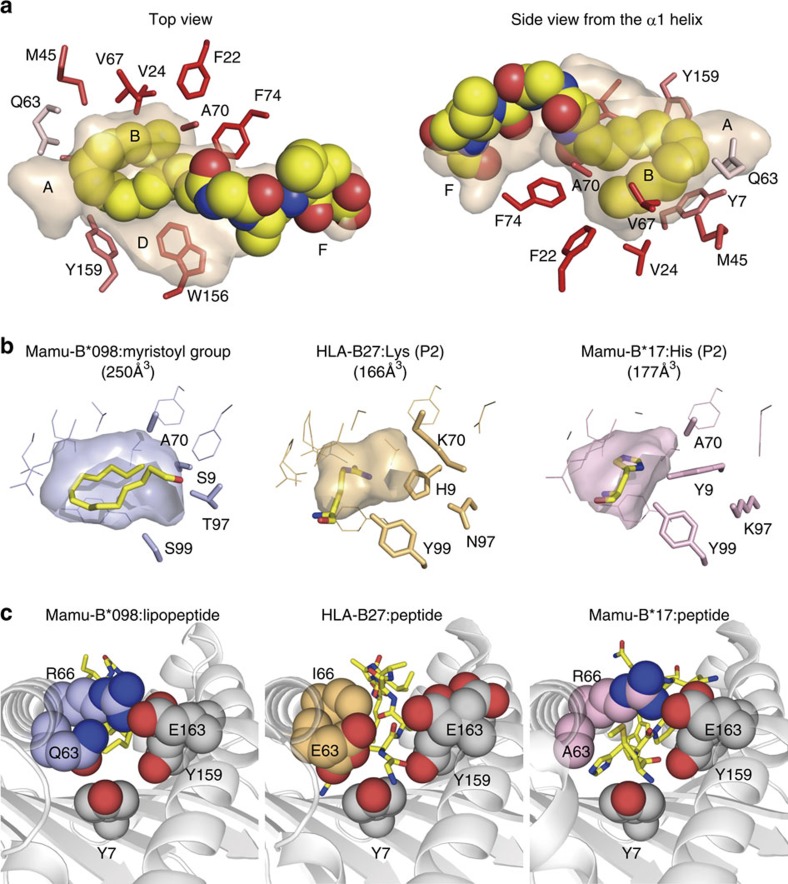
Interactions with the acyl chain of the lipopeptide. (**a**) C14nef5 is shown in yellow as a space-filling model with the semi-transparent Ag-binding groove of Mamu-B*098. The side chains of residues surrounding the acyl chain are shown in red. The intensity of the red colour correlates with the degree of hydrophobicity. (**b**) B pockets of Mamu-B*098 (blue), HLA-B27 (light orange) and Mamu-B*17 (pink) are shown as the semi-transparent groove and the side chains (lines) with emphasis on the non-bulky side chains (Ser9, Ala70, Thr97 and Ser99) of Mamu-B*098. C14 acyl chain or amino acid residues accommodated in each B pocket are displayed as yellow sticks. (**c**) A view from the A pocket points to a narrower channel between the A and B pockets in Mamu-B*098 (left) than in HLA-B27 (middle) and Mamu-B*17 (right). Key residues are presented as a space-filling model.

The acyl chain of C14nef5 did not gain access to the A pocket and was packed in a U-shaped configuration in the B pocket, which was lined with an array of hydrophobic or non-charged amino acid residues, including Tyr7, Phe22, Val24, Met45, Gln63, Val67, Ala70 and Phe74 (Fig. 3a). Numerous intermolecular van der Waals (VDW) forces and those established within the acyl chain supported its stable accommodation within the pocket (Supplementary Fig. 3 and Table 1). Besides its hydrophobic properties and low electrostatic potential (Supplementary Fig. 4), the Mamu-B*098 B pocket was also marked by its ample cavity (250 Å^3^), which was roughly 1.5-fold larger than that of the corresponding B pockets of other known MHC class I molecules (Fig. 3b). The side chains of His9, Lys70 and Tyr99 of HLA-B27, as well as those of Tyr9, Lys97 and Tyr99 of Mamu-B*17, protruded into the cavity; however, the amino acid residues at these positions (Ser9, Ala70, Thr97 and Ser99) were all small in Mamu-B*098, allowing the folded myristoyl group to fit in the B pocket (Fig. 3b).

The myristoyl group made a sharp U-turn instead of extending into the A pocket, thereby leaving the A pocket unoccupied, and the view from the A pocket towards the B pocket pointed to a highly constricted conduit between the two pockets (Fig. 3c, left panel). The bulky side chain of Gln63, as well as the side chains of Tyr7 and Tyr159, which were conserved among human and rhesus MHC class I molecules, protruded into the channel. Furthermore, the salt bridge that was established between Arg66 and Glu163 overhung the channel, making the opening as narrow as 4 × 4 Å^2^, a size that hardly allowed any peptide chains to pass through^15^. As a comparison, HLA-B27 had a bulky amino acid residue (Glu) at position 63, but lacked a salt bridge between positions 66 and 163 (Fig. 3c, middle panel), whereas Mamu-B*17 retained the salt bridge, but lacked the bulky residue at position 63 (right panel), thereby sustaining a structure that allowed the N-terminal residue of 9-mer peptides to gain access to the A pocket. The bulky side chains of Gln63 and Arg66 in Mamu-B*098 also contributed to the sidewall of the B pocket and established VDW interactions with the myristoyl group of bound lipopeptides (Fig. 3a and Supplementary Table 1). These structural features have not been reported in previously studied 9-mer peptide-presenting MHC class I alleles^12^ and we assumed that it may be difficult for Mamu-B*098 to bind conventional 9-mer peptides in a similar manner to that of other known MHC class I molecules.

### Accommodation of the C-terminal residue in the F pocket

**Figure 4:**
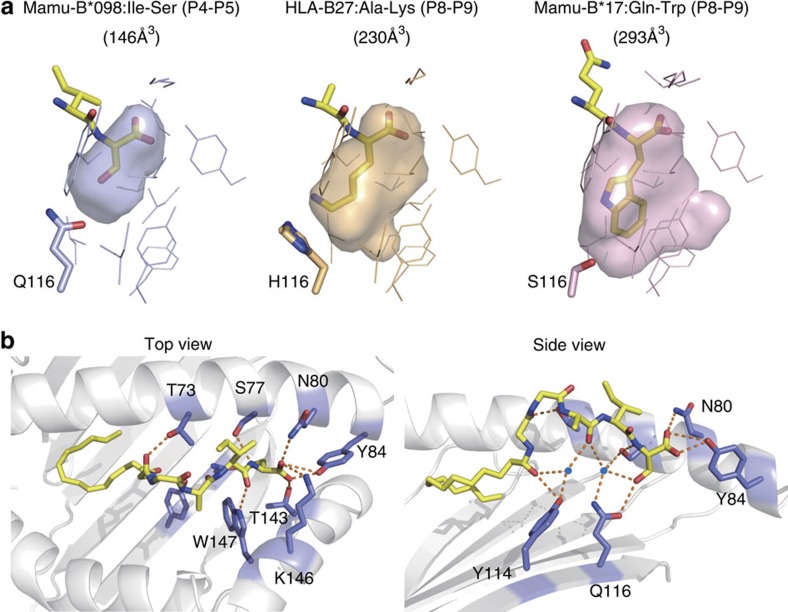
Interactions with the peptide chain of the lipopeptide. (**a**) F pockets of Mamu-B*098 (left, blue), HLA-B27 (middle, light orange) and Mamu-B*17 (right, pink) are shown as a semi-transparent groove and side chains (lines) with an emphasis on the key side chains at position 116. Two C-terminal amino acid residues of the ligands anchored in each F pocket are displayed as yellow sticks. (**b**) A hydrogen bond network was established between Mamu-B*098 and C14nef5. The top (left) and side (right) views of the Mamu-B*098:C14nef5 complex are displayed with hydrogen bonds (orange dashed lines). Water molecules are shown with blue spheres.

The C-terminal Ser residue (Ser5) of C14nef5 was anchored at the F pocket, which was smaller in size primarily due to the bulky side chain of Gln116 reducing the depth of the pocket (Fig. 4a). Thus, the C-terminal residue potentially accommodated in the F pocket is likely to be a relatively small amino acid residue, such as serine or threonine, constituting the N-myristoylation motif^16^, rather than the bulky amino acid residues found in most MHC class I-presented 9-mer peptides^17^. The main chain of Ser5 of C14nef5 was stabilized by a network of hydrogen bonds, involving Ser77, Asn80, Tyr84, Thr143 and Lys146, which is commonly found in peptide-bound MHC class I molecules^12^,^13^,^14^, whereas the side chain of Ser5 established a hydrogen bond with Gln116 (Fig. 4b).

### Spatial configuration of the bound lipopeptide

**Figure 5:**
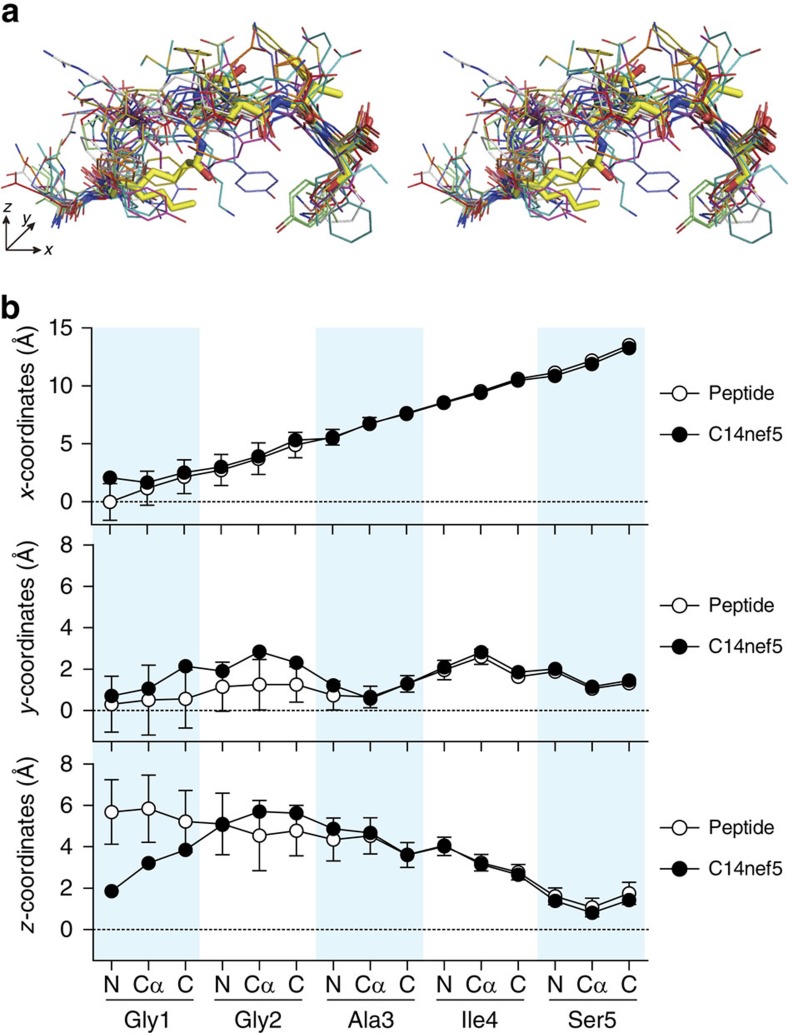
Spatial configuration of MHC class I-presented C14nef5 and conventional peptides. (**a**) A stereo view of C14nef5 (thick yellow sticks) bound to Mamu-B*098 overlaid with an array of representative 9-mer peptides (thin lines) bound to MHC class I molecules is shown. (**b**) The space coordinates of the main chain of MHC class I-bound peptides were determined for 280 registered MHC class I:peptide complexes and the mean values on the *x* axis (parallel to two α-helixes), *y* axis (vertical to the *x* axis and parallel to the line connecting the centres of two α-helixes) and *z* axis (vertical to the *x* axis and *y* axis) are shown with open circles. Error bars indicate s.d. The space coordinates of Mamu-B*098-bound C14nef5 were also determined and are shown with closed circles. It is worth noting that the three C-terminal residues (Ala3, Ile4 and Ser5) of C14nef5 and corresponding amino acid residues of conventional peptides exhibited a similar spatial configuration, whereas the two N-terminal residues (Gly1 and Gly2) of C14nef5 deviated in the *y*- and *z* axes from the position of the corresponding residues of conventional peptides.

Besides Ser5, the main chain of Ile4 of C14nef5 was stabilized by a hydrogen bond with Trp147 and the carbonyl oxygen of Ala3 made water-mediated hydrogen bonds with Tyr114 and Gln116 (Fig. 4b, right panel). As a result of these specific interactions, the spatial configuration of the three C-terminal amino acid residues (Ala3, Ile4 and Ser5) of C14nef5 bound to Mamu-B*098 was nearly identical to that of the corresponding residues of conventional 8- to 10-mer peptides bound to known MHC class I molecules (Fig. 5). In contrast, Gly1 of C14nef5 deviated significantly below the level of the corresponding amino acid residue (Fig. 5b, bottom panel). As the N-myristoylated Gly1 and C-terminal Ser5 residues were both buried deeply in the groove (Fig. 3c, right panel), this superimposed image also pointed to the primary role of the elevated middle three amino acid residues, in particular Gly2 and Ile4 with their side chains directed upwards, as T-cell epitopes.

## Discussion

The biology of MHC class I molecules represents one of the central foci for modern immunology research. Key findings obtained by pioneering studies, including the milestone discovery of the X-ray crystallographic structure of the ligand-bound HLA-A2 molecule, have established that MHC class I molecules bind 8- to 10-mer peptides and present them to specific CTLs^18^,^19^. The present study has provided functional and structural evidence to show that MHC class I molecules have evolved to include those that bind N-myristoylated short peptides and present them to specific CTLs. Therefore, the fixed concept of MHC class I-mediated presentation of long peptides to CTLs may need some modifications to incorporate a novel MHC class I function of lipopeptide Ag presentation.

The overall structure of Mamu-B*098 and the presence of six pockets in the Ag-binding groove were inherent in MHC class I molecules, whereas the large hydrophobic B pocket, small F pocket and the partially collapsed A pocket distinguished it from other MHC class I molecules that are known to bind long peptides. The myristoyl group of the lipopeptide adopted a U-shaped configuration and fit perfectly with the extended B pocket. Furthermore, inter- and intramolecular VDW forces established throughout the acyl chain synergistically reinforced its stable binding to the B pocket. These structural features suggested that the B pocket of Mamu-B*098 was optimally constructed, to accommodate a myristic acid, thereby providing reasonable explanations as to why the analogues of C14nef5, with either shorter (C6nef5 and C10nef5) or longer (C18nef5 and C22nef5) acyl chains, exhibit reduced or no antigenic activities^10^. Human CD1a molecules are known to bind the mycobactin-like lipopeptide JH-02215 by accommodating the acyl chain linked to the side chain of lysine in the A' pocket (Supplementary Fig. 5a)^20^; however, the A' pocket of CD1a and the B pocket of Mamu-B*098 differ significantly in their shape, size and position (Supplementary Fig. 5b). Indeed, amino acid residues that were predicted to establish the network of VDW interactions between CD1a and the acyl chain of JH-02215 do not appear to correlate with those of the interactions between Mamu-B*098 and the acyl chain of C14nef5 (Supplementary Fig. 5c). It remains to be addressed as to whether T cells may exist that specifically recognize C14nef5 in the context of CD1 molecules.

The F pocket of Mamu-B*098 also appears to be favourably used for binding N-myristoylated short peptides by taking advantage of the presence of the conserved small amino acid residue, either serine or threonine, at the C terminus of lipopeptides^16^. Most MHC class I-presented 9-mer peptides contain a bulky hydrophobic amino acid residue at the C terminus that is anchored at the F pocket; however, such common C-terminal residues may have been excluded from the small F pocket of Mamu-B*098. C14nef5 and its Ser to Thr mutant sustain virtually identical antigenic activities^9^, suggesting that the F pocket of Mamu-B*098 accommodates threonine and serine as well. As the myristoyl group and C-terminal serine/threonine are critical elements commonly found in proteins that undergo N-myristoylation^21^, Mamu-B*098 may have the potential to bind various naturally occurring N-myristoylated 5-mer peptides.

To the best of our knowledge, the spatially disconnected conduit between the A and B pockets of Mamu-B*098 has not been observed for any conventional 9-mer peptide-presenting MHC class I molecules. The A pocket typically contributes to stabilizing the binding of peptides by establishing a network of hydrogen bonds between their N-terminal amide group and the highly conserved tyrosine residues at positions 7 and 171 of MHC class I molecules^12^. Therefore, the lack of easy access to the A pocket indicates that Mamu-B*098 may be unable to bind conventional 9-mer peptides in a similar manner to that of known MHC class I molecules. Taken together, these structural features suggest that the Ag-binding groove of Mamu-B*098 has evolved to specifically bind N-myristoylated 5-mer peptides rather than conventional long peptides.

A soluble TCR αβ dimer derived from 2N5.1 interacted with C14nef5-loaded Mamu-B*098 molecules with a similar affinity to that for peptide-specific TCR–MHC interactions (Fig. 1d). Epitopic determinants for peptide-specific, MHC class I-restricted TCRs often involve as many as six or seven amino acid residues^1^,^22^,^23^. In contrast, the superimposed images of C14nef5 and conventional long peptides (Fig. 5a) indicated that the short stretch of C14nef5 amino acid residues (Gly2, Ala3 and Ile4) was only exposed externally with the potential to interact directly with specific TCRs. Furthermore, the three consecutive amino acid residues flanked by the conserved N-terminal glycine and C-terminal serine/threonine residues are unable to be randomly selected for protein N-myristoylation to occur naturally in cells^24^, suggesting that the diversity generated within this region may be more limited than that achieved in MHC class I-presented 9-mer peptides. Given that N-myristoylation occurs for viral and self proteins, this predicts the highly stringent negative selection executed in the thymus for the elimination of self lipopeptide-specific T cells; otherwise, autoimmune disorders may develop, as is occasionally observed in patients with viral infections^25^,^26^.

Despite such potential risks, the MHC class I-restricted CTL response that specifically senses the N-myristoylation of viral proteins may be beneficial to host defenses against viral infections. The N-myristoylation of the Nef protein is critical for anchoring it at the plasma membrane, thereby assisting its immunosuppressive activity. The N-myristoylated Nef protein was previously shown to downregulate the surface expression of MHC class I molecules, resulting in the inefficient induction of CTL responses to viral proteins^27^. The results of the present study indicated that this well-known evasive mechanism executed by HIV and SIV was simultaneously counterbalanced by the host immune system through eliciting CTL responses that precisely detected the N-myristoylation event of the Nef protein. As for a fraction of MHC class I molecules, including HLA-C proteins^28^, preliminary studies have suggested that the surface expression of Mamu-B*098 is not downregulated in Nef-expressing HeLa cells. Furthermore, cDNA clones with exon 6 selectively spliced out have been isolated from primary cells, which encode the Mamu-B*098 protein with the deletion of a long stretch of amino acid residues in the cytoplasmic domain, including tyrosine at position 320. As this residue is essential for Nef-mediated downregulation to occur^29^, we predicted that the surface expression of the truncated form of Mamu-B*098 may be unaffected by the Nef protein. Taken together, these findings point to a finely tuned balance achieved by the immunosuppressive activity of the N-myristoylated Nef protein and MHC class I-restricted CTLs capable of sensing the N-myristoylation event. Besides HIV and SIV, pathogenic viruses produce N-myristoylated proteins, which are often critical for pathogenesis^30^,^31^,^32^. The N-terminal amino acid residues constituting the N-myristoylation motif are hard for viruses to mutate without affecting protein function^33^,^34^. Therefore, N-myristoylated short peptide-presenting MHC class I alleles may be of evolutional significance.

## Methods

### Generation of monoclonal antibodies

The rhesus macaques (*Macaca mulatta*) used in this study were treated humanely in accordance with institutional regulations, and the experimental protocols were approved by the Committee for Experimental Use of Non-human Primates at the Institute for Virus Research and at the Primate Research Institute, Kyoto University. Rhesus macaque monocytes were isolated from PBMCs using MACS MicroBeads (Miltenyi Biotec, Bergisch Gladbach, Germany) conjugated with cross-reactive anti-human CD14 Abs. After immunizing BALB/c mice with these monocytes, spleen cells were obtained and fused with SP2/0 myeloma cells using the standard polyethylene glycol method^35^. Culture supernatants of the hybridoma clones were tested at a dilution of 1:1 for their reactivity to rhesus macaque monocytes by flow cytometry and positive clones were subjected to T-cell assays to assess blocking activities.

### Isolation of MHC class I genes

Total RNA was extracted from rhesus macaque monocytes using the RNeasy mini kit (Qiagen, Hilden, Germany) and first-strand cDNA was synthesized from 3 μg of RNA using the SMART cDNA Library Construction kit (Clontech, Palo Alto, CA), followed by a treatment with RNaseH (Invitrogen, Carlsbad, CA). DNA samples were incubated with 0.1 nmol of a biotinylated oligo-probe (5′-GAG GCC ACC CTG AGG TGC TGG GCC CTG-3′, specific for the conserved MHC class I α3 domain) for 5 min at 95 °C and then for 2 h at 55 °C, and probe-bound DNAs were captured by an incubation for 1 h at room temperature with 150 μg of Streptavidin–Dynabeads (Invitrogen). After magnetic separation, DNA was used as a template for PCR amplification. PCR was performed with the Advantage2 DNA polymerase (Clontech) according to the manufacturer's instructions. The DNA samples were treated sequentially with proteinase K and SfiI, followed by cloning into the pEXP-Lib vector (Clontech). Each of the isolated DNA clones was transfected into the rhesus macaque kidney epithelial cell line LLC-MK2 using the Lipofectamine 2000 reagent (Invitrogen) and the cell transfectants were used as Ag-presenting cells in T-cells assays with 2N5.1 as responder cells^36^.

### Generation of the C14nef5-loaded Mamu-B*098 complex

Recombinant proteins were prepared as described previously with some modifications^37^. Briefly, DNA constructs encoding the ectodomain of Mamu-B*098 (from Gly1 to Pro276 with the unpaired Cys167 mutated to Ser and a Met-Ala added to the N terminus) and rhesus β2m (from Ile1 to Met99 with a Met-Ala added to the N terminus) were synthesized and cloned into pLM1. The expression plasmids were introduced into the *E. coli* Rosetta 2 (DE3) pLysS strain (Novagen, Madison, WI) and colonies were inoculated into a Terrific Broth supplemented with 50 μg ml^−1^ ampicillin. Isopropyl-β-D-thiogalactoside was added to the culture at a final concentration of 0.5 mM when the OD_600_ value of the culture reached 0.5. After an additional 6-h culture, bacteria were harvested and lysed in a buffer containing 50 mM Tris-HCl pH 8.0, 1 mM EDTA, 0.1% NaN_3_, 10 mM dithiothreitol (DTT), 100 mM NaCl, 1% Triton X-100, 1% deoxycholate and 5 mM MgCl_2_, followed by a treatment with DNase I. After 6 h of stirring, the samples were centrifuged and the pellets were washed twice with a wash buffer containing 50 mM Tris-HCl pH 8.0, 1 mM EDTA, 0.1% NaN_3_, 1 mM DTT, 100 mM NaCl and 0.5% Triton X-100, and once with a rinse buffer containing 50 mM Tris-HCl pH 8.0, 1 mM EDTA, 0.1% NaN_3_ and 1 mM DTT. The purified inclusion bodies were then dissolved in a buffer containing 25 mM MES pH 6.0, 10 mM EDTA, 6 M Guanidine-HCl and 1 mM DTT, and insoluble material was removed by centrifugation. The supernatant was treated with 50 mM DTT for 3 h at 37 °C and aliquots were stored at −80 °C until use.

To obtain a trimer complex of B*098 heavy chains, β2m and C14nef5, the solubilized Mamu-B*098 heavy chains (33 mg) and β2m (12 mg) were refolded by rapid dilution in 1 litre of refolding buffer (100 mM Tris-HCl pH 8.3, 500 mM L-arginine, 2 mM EDTA, 0.5 mM oxidized glutathione and 5 mM reduced glutathione) containing 5 mg of C14nef5. After 48 h of continuous stirring at 10 °C, the samples were dialysed at 10 °C once against 4 l of 100 mM urea and twice against 4 l of 10 mM Tris-HCl pH 8.0. The refolded proteins were concentrated with DEAE-650M (Toyopearl, Tokyo, Japan) and subjected to HiLoad 16/600 Superdex 200 pg (GE Healthcare, Milwaukee, WI) size-exclusion chromatography and monoQ (GE Healthcare) anion exchange chromatography. The structural integrity of the protein complex was confirmed by native PAGE, circular dichroism spectra and enzyme-linked immunosorbent assay with conformation-dependent monoclonal antibodies to MHC class I (MB226) and β2m (MB217).

### Crystallization and structure determination

Crystals were formed at 20 °C by mixing 1 μl of a 10-mg ml^−1^ protein solution and 1 μl of a mother liquid containing 100 mM Tris-HCl pH 7.2, 2 mM zinc chloride and 13% PEG 6000, which was then cryoprotected in 20% ethylene glycol. Diffraction data were collected at 100 K (in a cold nitrogen gas stream) on a Rigaku Saturn A200 charge-coupled device detector (Rigaku/MSC, Woodlands, TX), using synchrotron radiation with a wavelength of 1.0 Å, at the BL26B1 station, SPring-8, Hyogo, Japan^38^. The resulting data set was processed, merged and scaled using HKL-2000 (HKL Research, Charlottesville, VA)^39^. The complex structure was solved by molecular replacement with Mamu-B*17 (PDB code 3RWJ) as a search model, as implemented in the CCP4i software suite^40^. The model was refined using the REFMAC5 (ref. 41) and PHENIX^42^ software packages. The structure was visualized and rebuilt using COOT 0.8.1 (ref. 43) and further modified on *σ*-weighted (2|*F*_o_|—|*F*_c_|) and (|*F*_o_|—|*F*_c_|) electron density maps. Zinc ions were carefully placed onto the structure by calculating its abnormal dispersion. Repeated processes of rebuilding and refinement resulted in *R*_work_/*R*_free_ values of 19.5 and 22.8% with 98.7% of residues being in favoured regions and 0.1% of residues being in outliers in a Ramachandran plot implemented in COOT. The four Mamu-B*098:C14nef5 complexes in the asymmetric unit were nearly identical (average r.m.s.d. of 0.37 Å over 276 Cα atoms) and the structure of one representative complex composed of chains D, E and F was described. Crystallographic images were produced using PyMOL (DeLano Scientific, San Carlos, CA).

The accessible surface area (Fig. 2e) and molecular contacts between C14nef5 and Mamu-B*098 (Supplementary Table 1) were analysed using CCP4i. The sizes of pocket B (composed of amino acid residues 7, 9, 22, 24, 45, 63, 66, 67, 70, 74, 97 and 99) and pocket F (composed of amino acid residues 77, 80, 81, 84, 95, 116, 117, 118, 123, 124, 143, 146 and 147) were calculated using the CASTp web server (http://cast.engr.uic.edu)^44^ with a probe radius of 1.4 Å (Figs 3b and 4a, respectively). The r.m.s.d. values over all the Cα atoms in the α1 and α2 domains after the superimposition of Mamu-B*098 with HLA-B27 (1K5N), Mamu-B17 (3RWG), HLA-A2 (3MRE), HLA-E (3BZE), HLA-G (3KYO), MR1 (4L4T), HFE (1A6Z) and CD1a_ENREF_38 (4X6F) were calculated (Fig. 2b). The spatial configuration of C14nef5 bound to Mamu-B*098 was compared with that of peptides bound to HLA-A1 (3BO8), HLA-A2 (3MRE), HLA-A3 (3RL1), HLA-A11 (1X7Q), HLA-A68 (4HX1), HLA-B8 (4QRS), HLA-B15 (1XR9), HLA-B27 (1K5N), HLA-B39 (4O2C), HLA-B44 (1M6O), HLA-B51 (1E27), HLA-B53 (1A1M), HLA-B57 (2BVP) and HLA-Cw4 (1QQD) (Fig. 5a). The VDW forces were estimated by a fragment molecular orbital calculation using the PAICS programme (Supplementary Fig. 3)^45^,^46^,^47^. Electrostatic potential was calculated using the eF-surf server (http://ef-site.hgc.jp/eF-surf/; Supplementary Fig. 4)^48^.

### Generation of β2 m-linked MHC class I proteins

Single-chain MHC proteins in which β2m was linked via a Gly-Ser linker (GGGGSGGSGSGGGSS) to the ectodomain of either Mamu-B*098 or Mamu-A*02, followed by a biotinylation sequence peptide (LHHILDAQKMVWNHR), were also expressed in *E. coli* and were purified in a similar manner to the trimer complex of Mamu-B*098, β2m and C14nef5. The β2m-linked Mamu-B*098 protein was refolded without C14nef5, whereas the β2m-linked Mamu-A*02 protein was refolded in the presence of the YY9 peptide (YTSGPGIRY). These recombinant proteins (3 mg) were biotinylated by being incubated for 12 h at 20 °C with 2 μg of BirA enzyme (Sigma-Aldrich, St Louis, MO) in a biotinylation buffer containing 100 mM Tris-HCl pH 8.0, 5 mM MgCl_2_, 4.5 mM ATP and 4 mM D-biotin, and were purified by size-exclusion chromatography.

### Generation of a soluble TCR dimer

TCR dimers were generated by the disulfide-linked TCR method^49^. DNA constructs encoding the extracellular domains of 2N5.1-derived TCRα (from Gln1 to Gly200 with Thr158 mutated to Cys) and TCRβ (from Gln1 to Asp242 with Ser169 mutated to Cys) were cloned into pET-21c(+), and the expression plasmids were expressed in *E. coli* as described above. Purified TCRα (15 mg) and TCRβ (16 mg) proteins were combined in 500 ml of a buffer containing 100 mM Tris-HCl pH 8.1, 400 mM L-arginine, 2 mM EDTA, 5 M urea, 3.7 mM cystamine and 6.6 mM cysteamine, and then incubated for 24 h at 10 °C with continuous stirring. The samples were dialysed three times against 4 l of 10 mM Tris-HCl pH 8.0 and purified as described above. A non-reducing SDS–PAGE analysis confirmed disulfide bond formation between the TCRα and TCRβ chains.

### T-cell assays

Streptavidin-coated 96-well plates (Thermo Scientific, Hudson, NH) were overlaid with 10 μg of the biotinylated β2m-linked Mamu-B*098 and Mamu-A*02 proteins, and lipopeptide Ags (1 μg) were loaded by an overnight incubation at 37 °C. The wells were then washed extensively and T cells (5 × 10^4^ per well) were added. After a 24-h incubation at 37 °C, aliquots of the culture supernatants were collected and the amount of IFN-γ released into the medium was measured by a human/monkey IFN-γ enzyme-linked immunosorbent assay kit (Mabtech, Nacka Strand, Sweden).

### Biolayer interferometry and SPR-binding assays

The binding of ligands to MHC class I molecules was analysed using the Octet RED96 system equipped with a streptavidin-conjugated biosensor (Pall ForteBio, Menlo Park, CA) according to the manufacturer's instructions. Biotinylated β2m-linked Mamu-B*098 and Mamu-A*02 proteins were immobilized on the sensor tip surface with a signal magnitude of ∼5.0 nm. The sensors were treated with 10 μg ml^−1^ biocytin for quenching, followed by incubation with lipopeptides (10 μM) in an assay buffer (20 mM Tris-HCl pH 7.5, 150 mM NaCl and 2% dimethylsulfoxide). After 45 min, the sensors were placed in the assay buffer without lipopeptides and incubated for an additional 30 min. Assays were performed at 30 °C. SPR-binding assays were performed to assess TCR interactions using the Biacore 3000 (GE Healthcare) according to the manufacturer's instructions. Lipopeptide Ag-loaded MHC molecules were immobilized on the sensor chip SA and then incubated at 30 °C with serially diluted 2N5.1 TCR proteins for 90 s (for association), followed by an incubation at 30 °C in an analyte-free assay buffer (20 mM Tris-HCl pH 7.5, 150 mM NaCl and 1 mM MgCl_2_) for 120 s (for dissociation). After reference subtraction, binding signals were analysed using the BIAevaluation 3.1 software. The equilibrium-dissociation constant (*K*_D_) was obtained by a global curve fitting method.

## Additional information

**Accession codes:** Atomic coordinates and structure factors for the reported crystal structure have been deposited in the Protein Data Bank under accession 4ZFZ.

**How to cite this article:** Morita, D. *et al*. Crystal structure of the N-myristoylated lipopeptide-bound MHC class I complex. *Nat. Commun.* 7:10356 doi: 10.1038/ncomms10356 (2016).

## Supplementary Material

Supplementary Information Supplementary Figures 1-5 and Supplementary Table 1.

## Figures and Tables

**Table 1:** Data collection and refinement statistics (molecular replacement).

	Mamu-B*098:C14nef5
*Data collection*	
Space group	*P*1
Cell dimensions	
*a*, *b*, *c* (Å)	46.40, 85.18, 127.99
*α*, *β*, *γ* (°)	89.20, 79.57, 90.02
Resolution (Å)	50–1.76 (1.79–1.76)
*R*_merge_	0.042 (0.532)
*I*/σ*I*	30.9 (2.2)
Completeness (%)	95.9 (78.1)
Redundancy	2.61 (2.5)
	
*Refinement*	
Resolution (Å)	1.76 (1.78–1.76)
No reflections	182443 (4351)
*R*_work_/*R*_free_ (%)	19.5 (30.3)/22.8 (31.9)
No atoms	
Protein	12621
MYR/EDO/TRS/zinc/chlorine	60/260/16/18/3
Water	810
B-factors (Å^2^)	
Protein	33.2
C14nef5	49.9
Water	36.6
R.m.s. d.	
Bond lengths (Å)	0.008
Bond angles (°)	1.11

EDO, 1,2-ethanediol; MYR, myristic acid; R.m.s.d., root-mean-square deviation; TRS, 2-Amino-2-hydroxymethyl-propane-1,3-diol.

Data were collected from a single crystal.

^*^The Highest resolution shell is shown in parentheses.
