# Supplementary material for: Crystal structure of the N-myristoylated lipopeptide-bound MHC class I complex
Source: Nat Commun. 2016 Jan 13;7:10356. doi: 10.1038/ncomms10356 (PMC4735555; doi:10.1038/ncomms10356)
Supplement: Supplementary Information — Supplementary Figures 1-5 and Supplementary Table 1. [file ncomms10356-s1.pdf]

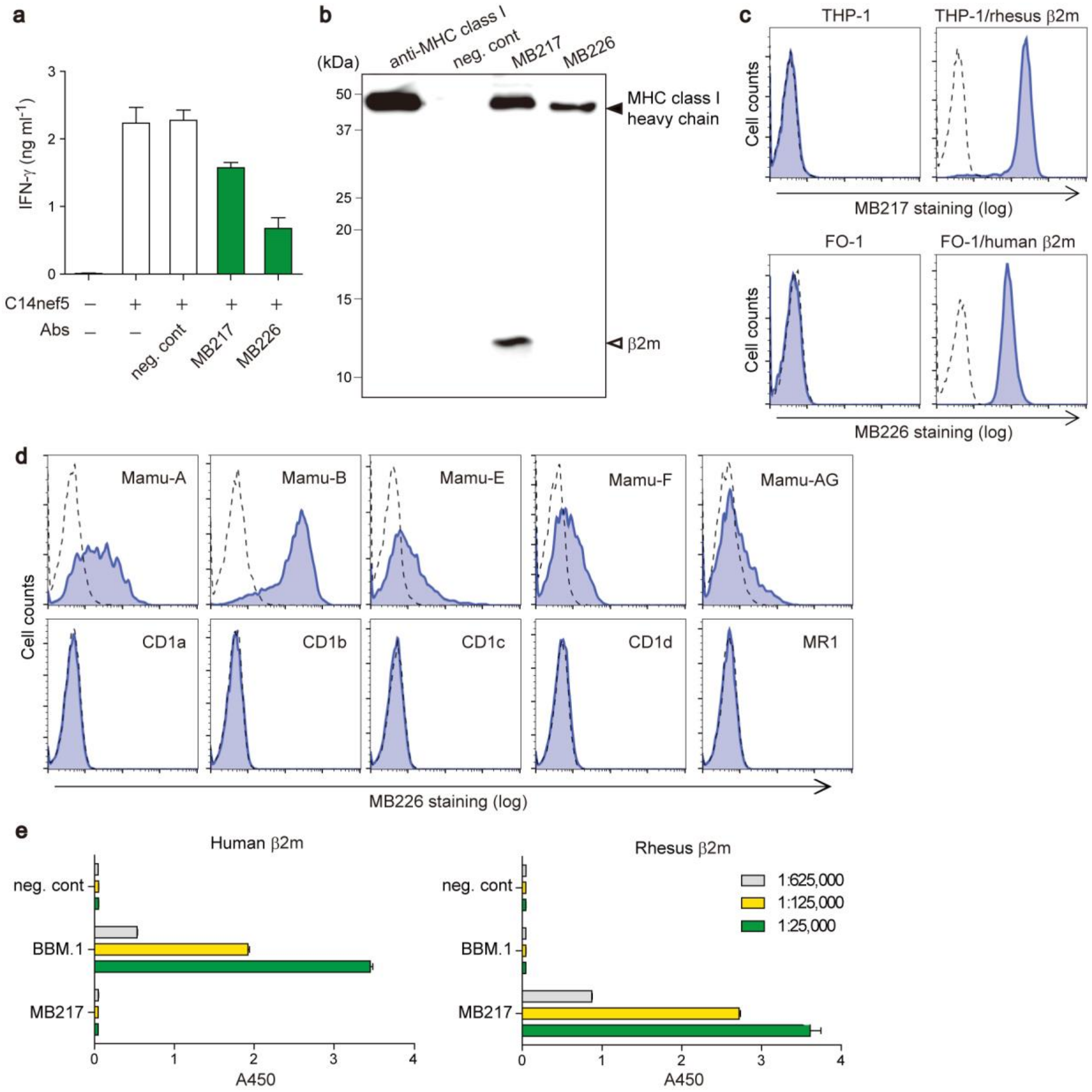

Supplementary Figure 1. Involvement of an MHC class I molecule in lipopeptide Ag-presentation. **a**, 2N5.1 cells responded to C14nef5 to produce IFN-γ in the presence of rhesus PBMCs derived from a positive donor, and the Ag-specific response was blocked by MB217 and MB226 mAbs (at a 1:400 dilution of ascites). **b**, Surface biotinylated LLC-MK2 cells were lysed in 0.5% Triton X-100, and immunoprecipitation was performed with the indicated mAbs. The 45 kDa species (MHC class I heavy chains) and 12 kDa species (β2m) immunoprecipitated by the Abs were indicated. **c**, MB217 failed to recognize the human monocytic cell line, THP-1, but reacted to rhesus β2m-transfected THP-1 cells (upper panels). MB226 failed to recognize the human β2m-deficient cell line, FO-1, but reacted to human β2m-reconstituted FO-1 cells (lower panels), indicating that MB226 cross-reacted to human

MHC class I molecules. Dotted lines indicate histograms with a negative control mAb. **d**, The mouse melanoma cell line, B16F10, was transfected transiently with the indicated genes, labeled with the MB226 mAb, and analyzed by flow cytometry. **e**, The reactivity of serially diluted MB217 ascites to plate-coated human (left) and rhesus (right) β2m was tested by ELISA. The reciprocal recognition of human and rhesus β2m by BBM.1 and MB217 was demonstrated. Experiments were performed in triplicate, and mean values with SEM are shown.

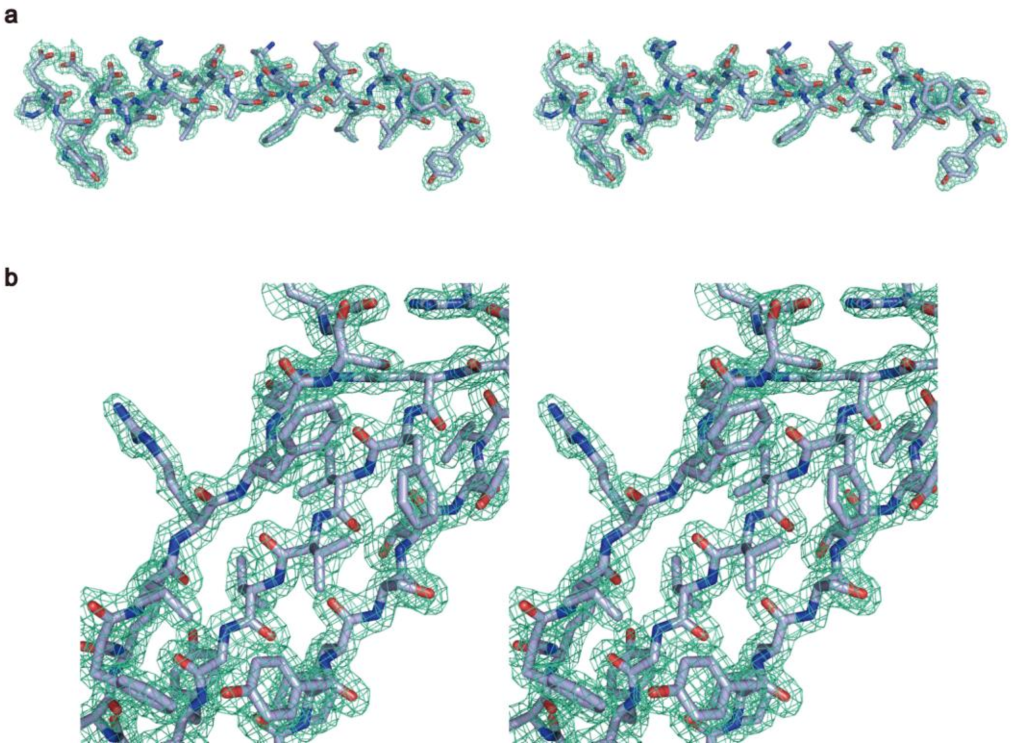

Supplementary Figure 2. Stereo images of a portion of the electron density map. Stereo views of α1 helix from Pro75 to Tyr85 (**a**) and β-sheets from Gly1 to Asp39 (**b**) of Mamu-B*098 with a 2Fo-Fc map (green mesh) contoured at 1.6σ.

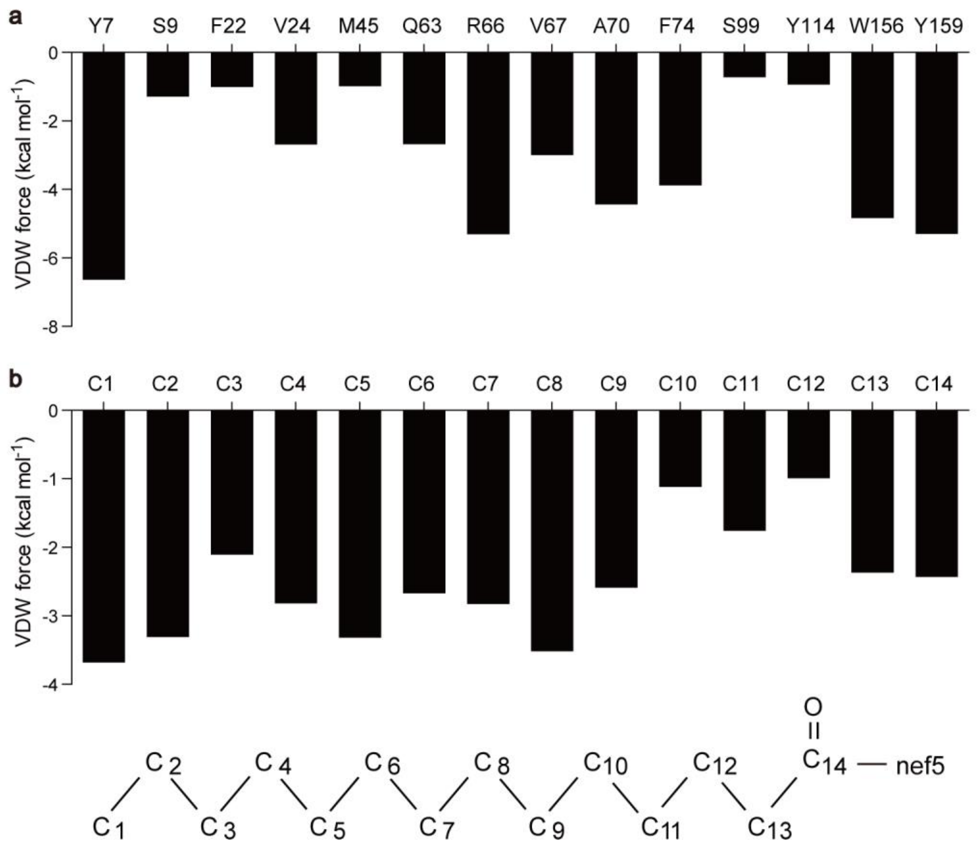

Supplementary Figure 3. VDW forces established between Mamu-B*098 and the acyl chain of C14nef5. The VDW forces involved in accommodation of the acyl chain were calculated for each amino acid residue of Mamu-B*098 (**a**) and for individual carbons of the acyl chain (**b**) by a fragment molecular orbital (FMO) method using the PAICS program. Only the amino acid residues of Mamu-B*098 with delta energy below the level of -0.5 kcal/mol are shown in **a**.

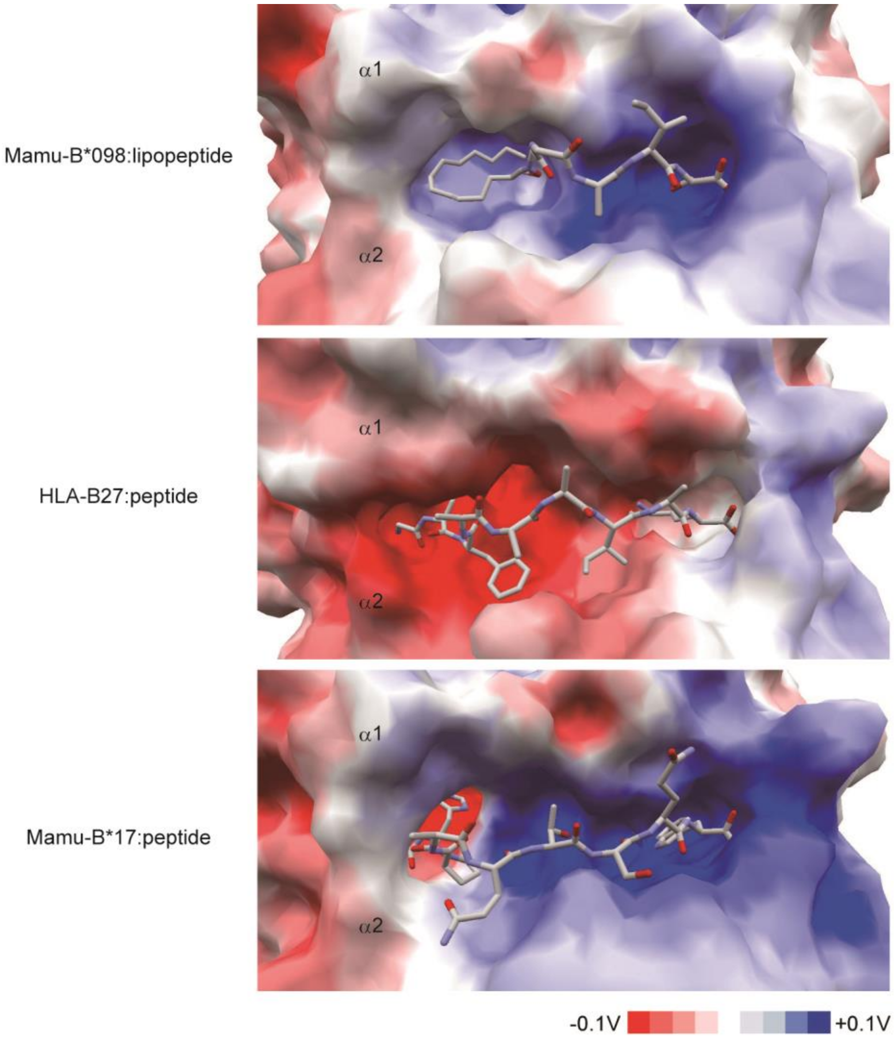

Supplementary Figure 4. Surface electrostatic potential of Mamu-B*098, HLA-B27, and Mamu-B*17. Surface electrostatic potentials of the Ag-binding groove are shown for Mamu-B*098, HLA-B27, and Mamu-B*17. Note the low electrostatic potential in the area involved in interactions with the acyl chain (top), which contrasted sharply with the high electrostatic potential detected in HLA-B27 (middle) and Mamu-B*17 (bottom).

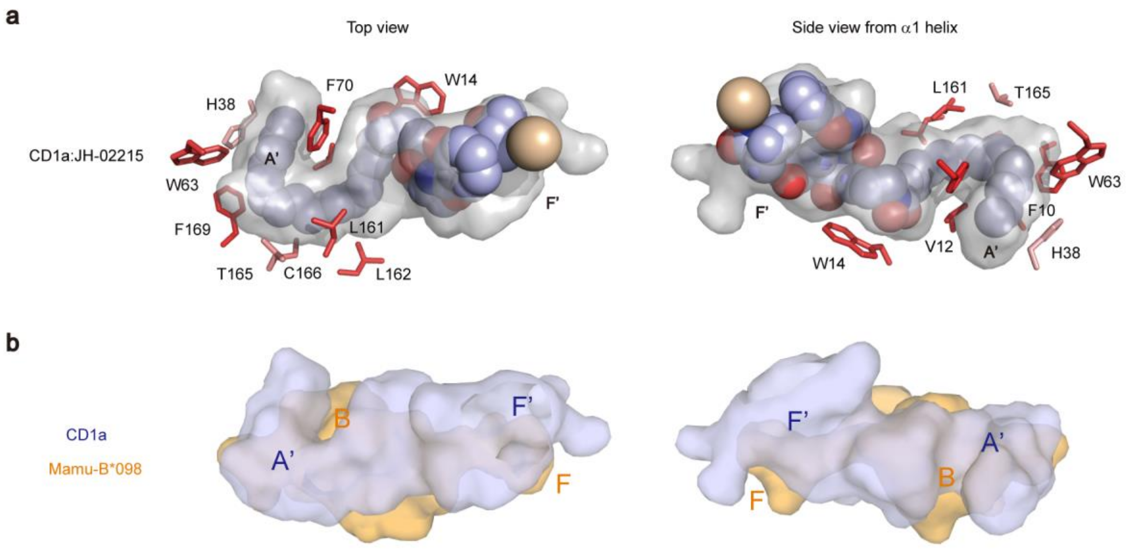

c

```
Mamu-B*098   1:GSHS--MRYFSTT-VSRPGRGEPRFIVVGYVDDTQFVRFDSDAASPKMEPRAPWMEQE-GPEYWEEQTRRVKDAAQTFRVSLGNLRGYYNQSE-A 90
Human CD1a   1:ADGLKEPLSFHVTWIASFYNHSWKQNLVSGWLSDLQTHTWDSNSST--IVFLCPWSRGNFSNEEWKELETLFR--IRTIR-SFEGIRRYAHELQFE 91
                           *   *          * *   * *    **          **       * * *          * * *    * *

Mamu-B*098  91:GSHTLQTMSGCDLGPDGRLLRGYYQQAYDGRDYIALNEDLRSWTAAD---EAAQNTQRK-WEAAGVAEQWRAYLEGECLECLRRYLENGKETLQRA 182
Human CD1a  92:YPFEIQVTGGCELH-SGKVSGSFLQLAYQGSDFVSFQNN--SWLPYPVAGNMAKHFCKVLNQNQHENDITHNLLSDTCPRFILGLLDAGKAHLQRQ 184
                    *   ** *   *       * ** * *         **        *                    *   *       *  **  ***

Mamu-B*098 183:EPPKTHVTHHPVSD-HEATLRCWALGFYPAEITLTWQRDGEDQTQDTELVETRPGGDGTFQKWGAVVVPSGEEQRYTCHVQHEGLP-EPLTLRW 274
Human CD1a 185:VKPEAWLSHGPSPGPGHLQLVCHVSGFYPKPVWVMWMR-GEQEQQGTQRGDILPSADGTWYLRATLEVAAGEAADLSCRVKHSSLEGQDIVLYW 277
                 *     * *        * *  ****       * * **  * *      *  ***        *  **     * * *  *      * *
```

Supplementary Figure 5. Interactions of the mycobactin-like lipopeptide, JH-02215, with human CD1a. a, JH-02215 is shown in blue as a space-filling model with the semi-transparent Ag-binding groove of human CD1a molecules (PDB code 1XZ0). The side chains of amino acid residues contributing to C-C contacts with the lipid portion of JH-02115 are shown in red. As in Fig. 3a, the intensity of the red color correlates with the degree of hydrophobicity. **b,** Superimposed images of Ag-binding pockets of human CD1a (blue) and Mamu-B*098 (orange) are shown. **c**, A ProbCons sequence alignment of Mamu-B*098 and human CD1a is displayed. The amino acid residues contributing to the C-C contacts are highlighted in orange. Asterisks indicate amino acid residues that are shared between the two molecules.

Supplementary Table 1. Contacts between C14nef5 and Mamu-B*098

C14nef5	Hydrogen bonds			C-C contacts
	Atom	Partner	Distance (Å)	
Myristoyl group	O1	Y114 (OH)	3.1	Y7, S9, F22, V24, Q63, R66, V67, A70, F74, W156, Y159, 10 intrachain interactions
		Water384	3.1	
Gly1	O	D69 (OD2)	2.9	None
		T73 (OG1)	2.7	
Gly2		None		T73
Ala3	O	Water139	3.0	T73, W147, V152
		Water384	3.3	
Ile4	O	W147 (NE1)	2.9	T73, V76, S 77, N80
Ser5	N	S77 (OG)	3.0	S77, L81, Y84, T143, K146, W147
	OG	Q116 (OE1)		
		Water139	2.8	
	O	Y84 (OH)	2.7	
		T143 (OG1)	2.7	
	OXT	N80 (ND2)	2.9	
		Y84 (OH)	3.3	
		K146 (NZ)	2.9	

* Contacts were assigned using a cut-off of 3.4 Å for hydrogen bond interactions and 4.4 Å for C-C interactions.
